# Cytoprotective and Antioxidant Effects of Hydrolysates from Black Soldier Fly (*Hermetia illucens*)

**DOI:** 10.3390/antiox12020519

**Published:** 2023-02-18

**Authors:** Kristian Riolo, Archimede Rotondo, Giovanna Loredana La Torre, Ylenia Marino, Gianluca Antonio Franco, Rosalia Crupi, Roberta Fusco, Rosanna Di Paola, Sabrina Oliva, Giuseppe De Marco, Domenico Savastano, Salvatore Cuzzocrea, Enrico Gugliandolo, Alessia Giannetto

**Affiliations:** 1 Department of Chemical, Biological, Pharmaceutical and Environmental Sciences, University of Messina, Viale F. Stagno d’Alcontres n. 31, 98166 Messina, Italy; 2 Department of Biomedical, Dental, Morphological and Functional Imaging Sciences, University of Messina, 98168 Messina, Italy; 3 Department of Veterinary Science, University of Messina, 98168 Messina, Italy; 4 Progetto Hermetia, Via Eritrea 10, 89013 Gioia Tauro, Italy

**Keywords:** protein hydrolysates, *Hermetia illucens*, bioactive compounds, antioxidant activity, Nrf2 activation, NMR analysis, quantitative gene expression

## Abstract

The black soldier fly (BSF), *Hermetia illucens,* has been recognized as one of the most promising insect species for its ability to valorize organic waste while producing a valuable larval biomass with a great potential as a sustainable source of nutrients, including proteins and bioactive molecules. In the present study, BSF larvae were used to produce and characterize the protein hydrolysates (BPHs) that were then evaluated for their potential biological activity in vitro. The BPHs obtained from the BSF larvae proteins by enzymatic digestion were characterized by Nuclear Magnetic Resonance (NMR) and polyacrylamide gel electrophoresis and assessed for their antioxidant activity (BPHs in the range of 0.1 to 1.5 mg/mL) in L-929 cells. Our findings show that BPHs can exert a dose-dependent cytoprotective role against H_2_O_2_-iduced oxidative stress in cells. This antioxidant activity relies on the reduction of ROS levels in challenged cells as measured by flow cytometry and fluorescence microscopy, together with the induction and nuclear translocation of Nrf2, as evaluated by qPCR and indirect immunofluorescence analysis, respectively. Overall, our findings on the remarkable biological activity of the BPHs obtained in a large-scale process strongly suggest the application of BPHs as ingredients promoting animal health in feed formulations.

## 1. Introduction

In the last few years, the world population growth has produced an excessive exploitation of food resources and the consequent need to find novel alternative proteins that can be used as a more sustainable nutritional source in animal feed. Among the new sources, insects have found considerable success due to their high protein content, ease of breeding, and low environmental impact. Indeed, the breeding of insects needs less space and less water and leads to lower emissions of methane and CO_2_ than livestock; furthermore, they have a greater feed conversion efficiency and reproductive capacity [1].

The black soldier fly (BSF), *Hermetia illucens*, is one of the insect species that is attracting increasing attention for its great potential as a new source of protein for animals and aquafeed [2,3]. Noteworthy is its ability to convert organic substrates into protein biomass, which is an important feature that can be applied for food waste valorization [4]. Behind the use of whole BSF larvae (BSFL) as flour for animal feed, the BSFL protein fraction subject to hydrolysis has demonstrated to have biological activities, such as antioxidant activities [5,6], and a potential ability to reduce allergenic risk [7]. Although antioxidants from plant sources are encouraged and being used, technological issues are arising due to diversity in the protein composition and matrices. Insects, including BSF, represent a valuable biotechnology incorporating the nutrients into their bodies during the bioconversion of organic matrices both reducing the amount of waste material and generating a more homogeneous and valuable biomass [8]. It is noteworthy that this ‘indirect’ biorefinery through insects can generate animal-based antioxidants, mainly proteins and peptides, with relevant nutritional value as compared to phenolic compounds, carotenoids, and vitamins from plants [9].

Recent studies have described food-derived bioactive peptides of animal origin with relevant bioactive potential; in particular, the protein hydrolysates from anchovy (*Engraulis encrasicolus*) have been used to evaluate antioxidant and anti-inflammatory potential in vitro (murine macrophages RAW 264.7 cells) and in vivo (mice, C57BL/6) [10]. ACE inhibitory activity was observed in spontaneously hypertensive male rats (SHRs) treated with protein hydrolysates of *Tenebrio molitor* larvae causing an antihypertensive effect [11]. Hydrolysates produced by *T. molitor* appear to be a promising alternative protein source for feeding sea trout [12,13]. 

Despite the great potential of BSFL proteins as a sustainable and alternative protein source to fishmeal in feed for the aquaculture sector, few data have investigated the ability of BSFL protein to provide valuable protein-derived sources with biological activity. The BSFL protein hydrolysates have shown potential as an alternative protein in rainbow trout (*Oncorhynchus mykiss*) and a better palatability in feed for Pacific white shrimp (*Litopenaeus vannamei*) that could be attributed to the high free amino acid content and water solubility [14,15]. The supply of a higher level of free dietary amino acids and peptides by BSF protein extract provided substrates for both protein synthesis-oriented metabolism and the associated energy requirement in fish, thus improving fish performance with increasing growth and feed efficiency. These findings showed the potential beneficial effects of using BSFL protein hydrolysates in animal feed [14]. 

Previous studies on protein hydrolysates from *H. illucens* centered mostly on different methods and factors affecting protein hydrolysate production, such as the enzyme used (trypsin, papain, bromelain, etc.) and the hydrolysis times [6,8]. Batish et al. [16] observed that the different enzymes used can give different results with alkalase leading to a higher degree of hydrolysis and a better antioxidant activity than pepsin. BSFL protein hydrolysates have been reported to have antioxidant activity, and it was observed that several processes in the production of hydrolysates, such as the killing methods of larvae (conventional drying, pasteurization, microwave drying, blanching, and freezing) or chemical action, can influence the antioxidant properties of BSF hydrolysates [17,18]. To date, the antioxidant activity in BSF protein hydrolysates has been evaluated by assays based on enzymatic models using chemical reactions relying on spectrophotometry. Mouithys-Mickala et al. [19] showed the ability of BSF derivatives to scavenge Reactive Oxygen Species (ROS) produced because of neutrophil activity. Although these chemical properties are documented, very few studies have investigated the potential biological effects of BSF protein hydrolysates in cells. 

To the best of our knowledge, data on the cellular response to BSFL protein hydrolysate exposure in challenged cells in vitro, evaluated both at the microscopic and molecular levels, are absent. This study aims at investigating the potential biological antioxidant properties of BSF larvae protein hydrolysates (BPHs), herein produced and characterized, by assessing their cytoprotective effects and the underlying cellular mechanisms in H_2_O_2_-challenged cells. Specific objectives concern the evaluation of the effects of BPHs on ROS scavenging activity and a better understanding of the mechanisms underlying the cytoprotective effects of BPHs by evaluating the possible involvement of the nuclear factor erythroid 2-related factor 2 (Nrf2), a specific transcription factor associated with the cellular response to oxidative stress. Data from this study may shed light on the potential role of these BSF protein hydrolysates in protecting against cellular oxidative stress. These findings could be useful for further applications in food and feed formulations as novel health-promoting ingredients obtained also from a system of food waste valorization in the context of a circular economy and reduction of environmental impact.

## 2. Materials and Methods

### 2.1. Materials and Chemicals

Protamex, Flavourzyme, and Alcalase were obtained from Novozymes China Inc., (Guangzhou, China). The solvents used in extractions and sample preparations (*n*-hexane, chloroform, and acetyl acetate) were of analytical grade and were purchased from Sigma-Aldrich (Milan, Italy) as were all the other chemicals (D_2_O, CDCl_3_, TSP(3-(Trimethylsilyl) propionic-2,2,3,3-d4 acid sodium salt), and sodium ortophosphate salts) used for the Nuclear Magnetic Resonance (NMR) analysis.

### 2.2. Insect Breeding

BSF larvae were provided by the established colony (www.progettohermetia.it, (accessed on 27 January 2023)) (Gioia Tauro, Italy). Four days post hatching, BSFL were reared on organic waste. The average composition of the substrate was 65% vegetal; 5% meat/fish; 25% bread/pasta/rice; and 5% other. After grinding, the substrate was opportunely treated and used to feed the larvae until harvesting. Larvae were collected, washed in sterile distilled water, pasteurized, and dried at room temperature (25 °C) prior to storing at −80 °C until analysis.

### 2.3. Protein Hydrolysate Production from BSF Larvae

BSFL were initially subjected to a defatting procedure and subsequent enzymatic digestion to produce BPHs. Briefly, defatting process was performed separately with the organic solvents *n*-hexane, chloroform, and acetyl acetate, as detailed below. Once the organic solvent was filtered, the residual solid was subjected to lyophilization and subsequent enzymatic digestion in water. This led to the solubilization of metabolites, which were later examined. These specific procedures are better detailed in the following Section 2.3.1 and Section 2.3.2. Preliminary, laboratory-scale tests were carried out to evaluate the possible influence of different defatting solvents on the recovered biological compounds especially in the perspective of the BPH production in a pseudo large-scale condition. BSFL were homogenized to achieve a mushy brown paste and later stored in a freeze-dry system to obtain the lyophilized powder.

#### 2.3.1. BSFL Water-Soluble Extract Production (Laboratory Scale)

A total of 3 mL of reagent-grade water and a defatting organic solvent (1:1 in weight) were added to 300 mg of the lyophilized BSFL powder. As organic solvents, *n*-hexane, ethyl acetate, and chloroform were used separately in different tests. The sample was sonicated at 700 W for 30 min and centrifuged at 2800× *g* at 15 °C to obtain two liquid phases bordered by an insoluble residual mud. The water-soluble phase was then directly analyzed by NMR or freeze-dried and frozen for further analyses. These preliminary analyses were performed to test the chance to readily extract both the hydrophobic and hydrophilic solutions by NMR. Although the chemical composition was not dramatically affected by different organic solvents in the small-scale process, our experimental evidence suggests that the *n*-hexane was, by far, the most suitable solvent for the next NMR analysis run on the water-soluble phase. Specifically, while chloroform was the best defatting solvent, health and environmental concerns do not suggest its use in large-scale processes; on the other hand, ethyl acetate, a solvent with minor environmental and ecological impacts, did not lead to a good extraction yield. Therefore, separation of the lipid component from BSF larvae was performed using *n*-hexane in the large-scale process to produce BPHs.

#### 2.3.2. Process Scale-Up 

The pasteurized and then frozen larvae (973 g) were ground and later lyophilized at −72 °C reaching a weight of 410.71 g. The lyophilized matter was extracted in *n*-hexane (1.6 L), and the solution was separated by a cumbersome filtration until a perfectly clear *n*-hexane solution without floating particles was obtained. *n*-hexane was moved away from the described solution through a rotary evaporator so that an orange buttery wax (83.4 g) was obtained; this wax was not considered in the present study. The insoluble matter recovered after the defatting procedure and filtration (described above) was lyophilized, weighted (276.27 g), and digested by using an enzyme mix (flavourzyme/protamex/alkalase 1:1:1) at 3% of the estimated solid weight (8.28 g) diluted in water in a final volume of 1.6 L, pH 6.9. The digestion was performed for 10 h at 50 °C, and finally, enzymes were inactivated at 90 °C for 40 min. The mixture was purified through sieves and later through a decanting, centrifugation, and separation routine. Final evaporation and lyophilization to estimate the water-soluble hydrolysate matter (126 g) were performed. 

### 2.4. Yield of BPHs 

The yield of protein hydrolysates can be calculated in different forms as it refer to the starting raw material, the dry matter, or the freeze-dried hydrolysate; for this reason, several quantifications were calculated using measures already reported elsewhere [6,20]. Any possible yield was based on the simple formula:

(1) $$Yield\left(\%\right)=\frac{m_{final}\left(g\right)}{m_{initial}\left(g\right)}\times 100$$

where *m_final_* is the mass of the final matter (i.e., freeze-dried BSF larvae protein hydrolysates), and *m_initial_* is the dry and fresh matter as indicated in Table 1.

### 2.5. Sodium Dodecyl Sulphate Polyacrylamide Gel Electrophoresis (SDS-PAGE)

Protein samples were quantified by the Pierce BCA Protein Assay Kit (Thermo Scientific) using a broad-spectrum Bovine Serum Albumin (BSA) standard curve and NanoDrop 2000 spectrophotometer (Thermo Fisher Scientific, Monza, Italy). Molecular weight distribution was evaluated by SDS-PAGE. Briefly, protein samples were incubated with sample buffer in a water bath (100 °C, 10 min) prior to being run on 15% separating gel. Electrophoresis was performed on a Bio Rad Mini-protean II apparatus. A prestained protein ladder of 10–250 kDa (Precision Plus Protein Standards Dual color, Biorad) was run together with protein samples. The polyacrylamide gel was stained with blue Coomassie solution (Coomassie brilliant blue R-200, 45% methanol, 45% water, and 10% glacial acetic acid) and rinsed in a destaining solution (45% methanol, 45% water, and 10% glacial acetic acid). Alliance LD287WL (UVITEC Ltd., Cambridge, UK) was used for acquiring gel images. 

### 2.6. NMR Analysis

A D_2_O solution (suitable as a gross frequency and field homogeneity reference) was prepared with 10 mM of TSP (3-(Trimethylsilyl) propionic-2,2,3,3-d4 acid sodium salt) used as NMR frequency (δ_1H_ = 0.0 ppm) and quantification standard and 500 mM PBS (phosphate buffer solution) with NaN_3_ (0.2%) to inhibit bacterial growth. This solution was added to the water-soluble samples in 1:9 volumetric ratios (measured pH 7.25 ± 0.05) in a commonly used practice [21,22,23]. All samples were analyzed at the same constant temperature (T = 298 K) with an NMR Agilent ProPulse 500 MHz spectrometer equipped with a ONE_NMR probe. The experimental set-up was carried out through the optimization of (1) the field homogeneity; (2) the 90° pulse duration to reach the maximum sensitivity (6.8 ± 0.1 ls; power attenuation 60 Db); (3) the water presaturation frequency with power at 25 Hz; and (4) the suitable acquisition time and time delay according to the relaxation times (T1). This last parameter was measured through the “inversion recovery” experiment and validated by the constant integration ratios after a different number of scans. Because T1 were up to 3 s, 2 s of acquisition time and 13 s of time delays were chosen. For any sample, three different analytical experiments were performed evidencing different features: (1) standard water-presaturated ^1^H experiment; (2) water presaturation during an 1d ^1^H-NOESY sequence with a very low mixing time called “NOESYpresat”; and (3) water-presaturated ^1^H experiment with a spin-echo delay able to filter out the high-molecular-weight-molecule “pr_cp” experiment. The detection and quantification of free α-amino acids and other metabolites were carried out by the MARA-NMR quantification, which was already applied successfully in the previously mentioned studies [21,22,23,24]. The presence of several metabolites was confirmed through the consistency of 1D TOCSY experiments, selecting the different spin systems representing diverse molecular systems. Details about assignments and integrations are available in the Supplementary Materials (Figure S1 and Table S1).

### 2.7. Cell Culture 

The murine fibroblast L-929 cells were cultured in DMEM including 10% fetal bovine serum streptomycin (100 μg/mL) and penicillin (100 μg/mL) at 37 °C with 5% of CO_2_. Once the cells reached about 80% confluence, they were harvested and subcultured after trypsin/EDTA treatment. 

### 2.8. Cell Viability Assay

Cell viability assay was tested through the 3-(4,5-dimethylthiazol-2-yl)-2,5-diphenyl-2H-tetrazolium bromide (MTT) assay as previously described [25]. Briefly 1 × 10^4^ cells were seeded in 96-well plate and incubated for 24 h at 37 °C and 5% CO_2_. Cells were incubated with different concertation of BPHs (50, 10, 5, 1.5, 1, 0.5, 0.1, 0.05, and 0.01 mg/mL) for 24 h. Then, cells were incubated with MTT solution (0.5 mg/mL, Invitrogen,) for 2 h at 37 °C. Subsequently, MTT solution was removed, and 200 μL of DMSO was added, and absorbance was recorded at 540 nm with a microplate reader (Multiskan, Thermo Fischer Scientific) with Skanlt Software 6.0.2 (Thermo Fisher Scientific, Italy). To test the cytoprotective effect of BPHs in the presence of H_2_O_2_, cells were treated in 96-well plate with the noncytotoxic concentrations of BPHs for 2 h; then, H_2_O_2_ 1 mM was added, and cells were incubated at 37 °C for 24 h. After incubation period, MTT assay was performed as described above. The results were determined in percentages of the viable cells compared to the untreated control.

### 2.9. Determination of Intracellular ROS

The generation of intracellular ROS was determined by carboxy-H2DCFDA (Invitrogen, Thermo Fisher #C400) assay according to the manufacturer’s protocol. Briefly, L-929 cells treated with BPHs (1.5, 1, 0.5, 0.1, and 0.05 mg/mL) for 2 h. Then, 10 μL of H_2_O_2_ (1 mM) was added for 1 h. Fluorescence intensity signals were evaluated with Attune NxT cytometer (Thermo Fischer Scientific) and under fluorescence microscope (Evos M5000, Thermo Fisher) [26]. All experiments were repeated three times independently. 

### 2.10. Nrf2 Subcellular Localization Assay

L-929 cells were seeded in 12-well plates and incubated with BPHs (1.5, 1, 0.5, and 0.1 mg/mL) in presence or not of H_2_O_2_ (1 mM) for 24 h. Cells were fixed in 4% paraformaldehyde in PBS and 0.1% Triton X-100 solution in PBS and then incubated with anti-Nrf2 produced in rabbit (SAB4501984, Sigma-Aldrich) followed by antirabbit IgG conjugated with Texas Red (#T-6391, Thermos Fisher). For nuclear counterstaining, cells were stained with DAPI (#D3571, Invitrogen Thermo Fisher). Fluorescent images were captured on EVOS M 5000 (Thermo Fisher). 

### 2.11. mRNA Levels of nrf2 

Total RNA was extracted from cells using TRIsure reagent (Bioline, France) and assessed for RNA quality and quantity by 1% (*w*/*v*) agarose gel electrophoresis and NanoDrop 2000 spectrophotometer (Thermo Fisher Scientific, Italy). After removing any potential genomic DNA contamination, cDNA synthesis was performed from 1 μg total RNA using the QuantiTect reverse transcription kit (Qiagen) following manufacturer’s instructions. The transcript levels of *nrf2* were quantified by quantitative Polymerase Chain Reaction (qPCR) using the QuantiTect SYBR^®^Green PCR Kit (Qiagen) in a Rotor-Gene Q2 plex Hrm thermocycler (Qiagen). cDNA samples (1:50 diluted) were amplified using gene-specific qPCR primers for target gene (*nrf2*F: TTTCAGCAGCATCCTCTCCA and *nrf2*R: AGCCTTCAATAGTCCCGTCC) and for reference genes (*gadph*F: TCCATGACAACTTTGGCATTG; *gadph*R: TCACGCCACAGCTTTCCA; *36b4*F: GGACCCGAGAAGACCTCCTT; and *36b4*R: GCACATCACTCAGAATTTCAATGG). Biological replicates (*n* = 6) were run in duplicate together with minus reverse transcriptase and no template controls for each reaction. PCR efficiency and specificity were evaluated as previously described [27]. The normalization factor calculated by the GeNorm Software from the two most stable reference genes (*gadph* and *36b4*) was used to correct the raw data as reported by Nagasawa et al. [28]. 

### 2.12. Statistical Analysis

All statistical analyses were performed using the GraphPad Prism version 9 software (GraphPad Software Inc., La Jolla, CA, USA). Data from in vitro studies were evaluated for differences among groups by one-way ANOVA followed by Bonferroni’s post hoc test for multiple comparisons. Differences in transcript levels of tested genes among the experimental groups were assessed by analysis of variance followed by Student–Newman–Keuls post hoc tests. A *p*-value less than 0.05 was considered statistically significant.

## 3. Results

### 3.1. Production and Yield of Protein Hydrolysates from BSF Larvae 

Defatting using *n*-hexane and subsequent enzymatic digestion produced 126.40 g of water-soluble powder from BSF larvae (973 g fresh weight). This represented 30% of the dry matter, and consistently, the NMR quantification of all the metabolites in the water solution was referred to as 29.7% of this same reference. NMR quantification, unlike the other targeted techniques, also allows the detection and quantification of nonamino acid metabolites; therefore, it was possible to know the neat presence of free amino acids (FAAs, 20%) with respect to the starting dry material. Table 1 summarizes all the meaningful values inferred by our large-scale experiments.

As noted in paragraph 2.6, the yield can be calculated in different ways; moreover, the NMR analysis distinguished the other metabolites from the free amino acids (FAAs) so that it was possible to infer the yield concerning just the aminoacidic composition.

### 3.2. BPH Molecular Weight Distribution 

**Figure 1:**
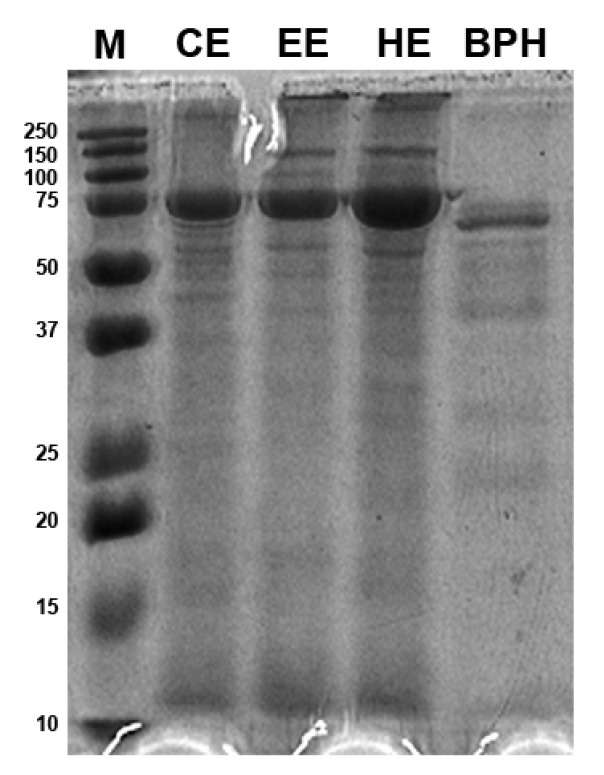
Protein patterns of black soldier fly (BSF) protein extracts and BSF protein hydrolysates (BPHs) resolved by Sodium Dodecyl Sulphate Polyacrylamide Gel Electrophoresis (SDS-PAGE). M, marker; CE, chloroform extract; EE, ethyl acetate extract; HE, hexane extract; BPHs, BSFL protein hydrolysates.

The molecular weight distribution of BSF larvae protein extracts and protein hydrolysates was estimated by SDS-PAGE (Figure 1). The protein patterns obtained by the three different protein extraction methods, i.e., organic solvent extraction with *n*-hexane, ethyl acetate, and chloroform, did not show relevant differences in band distribution and appeared in a molecular size range of 10–150 kDa with a strong signal at 75 kDa that has the highest intensity in the *n*-hexane extract. Moreover, bands larger than 250 kDa were also present. The BPHs, instead, showed most protein bands in the narrow range of 10–75 kDa; the 75 kDa band had a thinner appearance as compared to the insect protein extract samples. The hydrolysate patterns presented most bands in the range of 50–75 kDa, while isolated bands between 25 and 37 kDa and between 20 and 25 kDa and at 10 kDa were also revealed.

### 3.3. NMR Data

The identified metabolites and quantification results are reported in Table 2 as extracted by the NMR analysis, excluding a few spectral regions where the spin-echo delay (“pr_cp” experiment) showed remarkable differences labelling high-molecular-weight molecules. Three identical mother samples and the three corresponding 1:10-diluted solutions were analyzed in order to confirm the quantification within the standard deviation after at least six measurements. The quantification concerned 16 FAAs and 14 other metabolites giving a total panel of 30 species. This protocol is indeed extendable to the relatively quick monitoring of flows spiked from BPH bioreactors.

### 3.4. Effects of BSF Protein Hydrolysates on L-929 Cell Viability In Vitro

**Figure 2:**
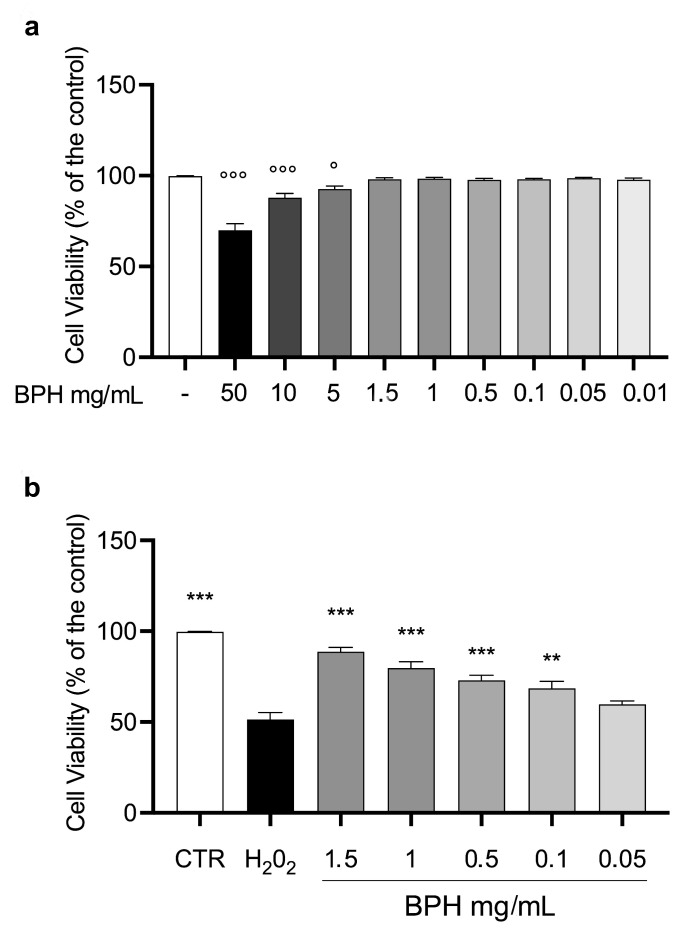
L-929 cell viability. (**a**) L-929 cell viability after 24 h of BPH exposure. (**b**) BPH protective effect against H_2_O_2_-induced cytotoxicity in L-929 cells. Data (mean ± SE) are representative of at least three independent experiments. °°° *p* < 0.001 vs. CTR; ° *p* < 0.05 vs. CTR; *** *p* < 0.001 vs. H_2_O_2_; ** *p* < 0.01 vs. H_2_O_2_.

To evaluate the effect of BPHs on cell viability, L-929 cells were incubated with different concentrations of BPHs (50 to 0.01 mg/mL) for 24 h. After the incubation time, the MTT test showed that from the dose of 5 mg/mL to 0.001 mg/mL, the % of cell viability was almost comparable to the control group (Figure 2a). Based on the results mentioned above, the cytoprotective and antioxidant effects of BPHs were evaluated in the L-929 cell challenged with H_2_O_2_. Cells exposed to H_2_O_2_ showed a significant reduction in cell viability compared to the control group (Figure 2b). Interestingly, pretreatment with BPHs in the range of 0.1 to 1.5 mg/mL determined a significant improvement of % of cell viability compared to the H_2_O_2_ group showing a cytoprotective effect of BPHs at 1.5, 1, 0.5, and 0.1 mg/mL in the challenged cells.

### 3.5. In Vitro Effects of BPHs against ROS Production in L-929 Cells

**Figure 3:**
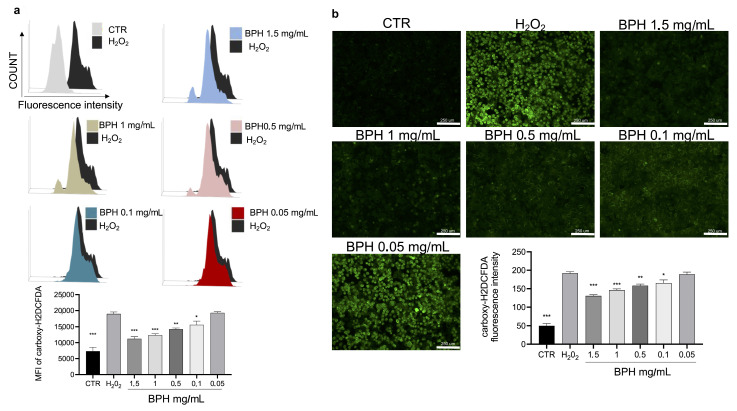
(**a**) Flow cytometry analysis of carboxy-H2DCFDA probe fluorescence of each experimental group compared to H_2_O_2_ group. (**b**) Fluorescence microscopy images of carboxy-H2DCFDA positive cells for each experimental condition. Data (mean ± SE) are representative of at least three independent experiments. *** *p* < 0.001 vs. H_2_O_2_; ** *p* < 0.01 vs. H_2_O_2_; * *p* < 0.05 vs. H_2_O_2_.

The cytotoxic activity of H_2_O_2_ is related to the increase in cellular oxidative stress and ROS. To evaluate the protective effect of BPHs on H_2_O_2_-induced oxidative stress, the intracellular ROS was stained by a carboxy-H2DCFDA probe. Carboxy-H2DCFDA fluorescence staining showed that H_2_O_2_ exposure significantly increased ROS levels, compared to the untreated cells (Figure 3a,b). Notably, the treatment with BPHs significantly reduced the increase in ROS levels induced by H_2_O_2_; BPHs at the highest concentrations (1.5 and 1 mg/mL) showed comparable effects on ROS levels against H_2_O_2_; in cells treated with BPHs at 0.5 and 0.1 mg/mL, a significant dose-dependent protective effect against H_2_O_2_ was observed (Figure 3a,b).

### 3.6. Effect of BPHs on Nrf2 Activation

**Figure 4:**
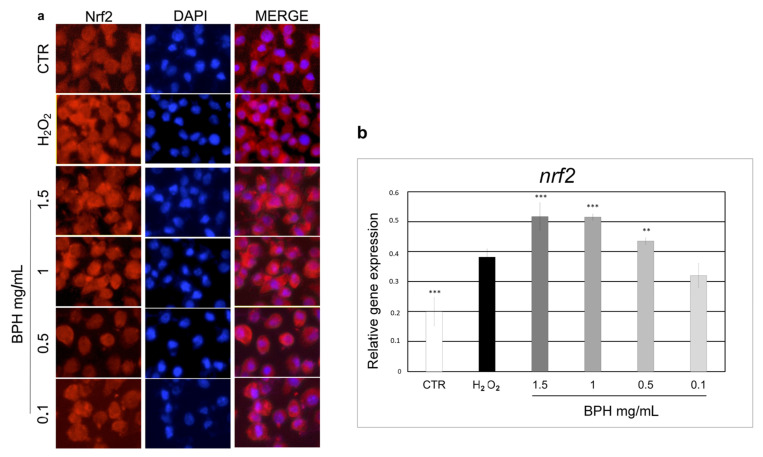
(**a**) Indirect immunofluorescence for Nrf2 expression and subcellular localization. For nuclear counterstaining, cells were stained with DAPI. (**b**) Relative mRNA levels of *nrf2* in L-929 cells treated with BPHs in each experimental group. Data are expressed as mean ± S.D. (*n* = 6). *** *p*< 0.001 vs. H_2_O_2_; ** *p*< 0.01 vs. H_2_O_2_.

The activation of the transcription factor Nrf2 has been shown to be able to reduce the ROS generation, thus gaining further insights into the mechanism underlying the BPH-mediated cytoprotective effect. As shown in Figure 4, Nrf2 localization and expression were analyzed by immunofluorescence staining and qPCR, respectively. Immunostaining of Nrf2 in L-929 cells showed an increase in Nrf2 expression and nuclear translocation upon BPHs 1.5, 1, 0.5, and 0.1 mg/mL treatment (Figure 4a). These findings were confirmed by qPCR analysis (Figure 4b) where the *nrf2* mRNA levels in cells treated with BPHs at 1 and 1.5 mg/mL were 1.4 times higher than the H_2_O_2_ group, while cells treated with 0.5 mg/mL BPHs were 0.8 times higher than the H_2_O_2_ group; no statistical differences were found between the 0.1 mg/mL BPH and H_2_O_2_ treatments, showing a dose-dependent gene expression trend. These findings confirm the observed cytoprotective effect of BPHs against oxidative stress induced by H_2_O_2_ and show that BSH can modulate the activity of Nrf2 to counteract oxidative stress. 

## 4. Discussion

Worldwide, insect proteins are recognized as sustainable alternatives to address the global protein shortage due to the increasing human population. The sustainability of BSF farming and the valuable nutritional properties of BSF larvae have encouraged the research on the potential applications of BSF protein in food and feed formulation [1]. In addition to the well-documented importance as a sustainable source of protein for animal feed, BSF larvae proteins have been demonstrated as a valuable source of bioactive molecules with biological activities [29]. Enzymatic hydrolysis represents a well-validated procedure to produce small-sized peptides, as well as free amino acids with improved biological activities as compared to proteins with potential anti-inflammatory, antioxidant immunomodulation, and antibacterial properties [6,30,31]. More recently, protein hydrolysates from BSF have been produced and characterized for their amino acid composition and their direct ROS scavenging activity [5,6]. 

However, to the best of our knowledge, no data on the cytoprotective effects, evaluated both at the microscopic and molecular levels of BPHs obtained by enzymatic hydrolysis, are available on the cellular system nor the underlying mechanisms in protecting against ROS-induced cytotoxicity. In the present study, protein hydrolysates were produced from BSF larvae by defatting followed by enzymatic hydrolysis. Following BPH chemical characterization and molecular distribution analysis, the obtained BPHs were evaluated for their antioxidant properties in L-929 cells. The preliminary small-scale procedure of BPH production gave the necessary background information for the process scale-up that can be useful in the perspective of future applications of BSF protein hydrolysates in diverse industrial fields.

The chemical extractions and digestion were basically performed until the total decomposition of the macromolecules to simple molecules and free amino acids (FAAs). The yield of the three extracted fractions (the fat fraction extracted by *n*-hexane; the hydrolysate from the water-soluble fraction rich in aminoacidic matter; and the insoluble remaining solid containing chitins and chitosans) is comparable to the results reported elsewhere [5,32]. The obtained water-soluble fraction was subjected to enzymatic digestion to produce protein hydrolysates for evaluating their potential biological activity. Indeed, protein derivatives (i.e., key peptides in the hydrolysates, FAAs, and other small water-dissolved molecules) are known to be characterized by higher biological activity as compared to protein, while maintaining their nutritional value [19]. In animals, digestive processes as well as peculiar metabolic cellular processes can influence the biological activity of protein and peptides by ubiquitous or species-specific cellular mechanisms that in turn modulate the bioactivity of the ingested proteins [14]. 

The characterization of the BSFL proteins and their hydrolysates by SDS-PAGE analysis showed that the protein patterns were not influenced by the three organic solvents used for protein extraction. In the perspective of the large-scale production of BPHs, the utilization of a green solvent, such as ethyl acetate for protein extraction, should be considered for future applications. The molecular weight distribution analysis showed that the hydrolysis reaction produced a protein pattern characterized by several bands below 25 kDa that is considered the peptide fraction [6]. The protein bands in the molecular weight in the range of 25–37 kDa could belong to proteases used to catalyze the hydrolysis reaction as already found by Firmansyah and Abduh [6]. In general, differences in the molecular distribution profile of our BPHs in respect to previous studies are probably due to different proteases and conditions (i.e., pH and time of hydrolysis) that can hydrolyze BSF proteins into small peptides and amino acids with different efficiencies [5,6,17]. 

The metabolite determination and quantification results are recovered mainly from the NOESYpresat experiments provided that other used NMR techniques (simple presat and cp_pr) did not show significant differences in the spectral regions used for integration. The main value of the NMR technique is the holistic profiling of the samples without any chemical modification or functionalization. This allows the simultaneous quantification of 30 species including 16 FAAs (15 alpha amino acids and 4-amino butyrate), together with organic acids, amines, and other chemicals. The chemical profile is comparable to BSF extracts obtained by other studies [33]. It was already observed by NMR that the BPH composition changes according to the different killing methods or metabolic phenotypes, and our results are consistent with the quick pasteurization used to kill larvae before the freezing process (our unpublished data also show the differences in the case of slowly frozen larvae) [34,35]. The metabolite profile of BPHs evidenced that among the identified amino acids (e.g., leucine, isoleucine, valine, threonine, alanine, proline, glutamate, methionine, aspartate, serine, phenylalanine, tryptophane, tyrosine, glycine, and 4-aminobutyrate), alanine and glutamate were the most represented nitrogen compounds solubilized by enzymatic hydrolysis, and these data are consistent with previous findings regarding the nutritional values of BSF [4,36]. Furthermore, the amount of valine, isoleucine, and leucine was higher than in the BSF larvae fed poultry manure and other vegetable waste but was compatible with organic food waste [20,37,38]. Beyond the detection and quantification of amino acids, the NMR analysis allowed the identification of several metabolites (e.g., ethanol and formate, choline, betaine, uracil, and amines also bioactive as tyramine); this whole chemical panel provides an insight into the biochemical BPH characteristics. As Ho et al. [35] suggested, the presence of ethanol and formate can be related to fermentation processes due to anaerobic microorganisms. 

The detection of 3-hydroxy propionate (3-HPO), trimethyl amine (TMA), and dimethylamine (DMA) can be due to biological degradation processes. Indeed, TMA and DMA have been shown to result from protein degradation by BSF gut microbiota [39]; on the other hand, 3-HPO is often implied in bacterial pathways involving its isomer lactate and glycerol [40], which are known to be always present in BPHs.

Recently, many studies have demonstrated that peptide hydrolysates from insects possess several biological activities [41]. In particular, recent studies highlighted the biological potential of peptide derivatives from black soldier fly *H. illucens* suggesting an important antioxidant potential for these derivatives where the characteristic protein and peptide composition contributes to the important biological activity and antioxidant potential [6,16,42]. Due to the lack of scientific data on the biological properties of hydrolysates from *H. illucens* to date, our first aim was to test in vitro the effect of BPHs. Thus, the well-validated in vitro model of H_2_O_2_-induced oxidative stress was used in L-929 cells. Our results show that BPH treatment had a significant cytoprotective effect against the H_2_O_2_-induced reduction of cell viability. The reduction in cell viability induced by H_2_O_2_ is related to the increase in cellular oxidative stress and ROS generation. Our results show that BPH treatment was able to significantly reduce the intracellular ROS generation induced by H_2_O_2_ exposure. This can be explained both by a scavenger action on ROS but also by the improvement of endogenous cellular defenses. Intracellular ROS control is reached when there is a balance between ROS generation and ROS scavenging by both enzymatic and nonenzymatic antioxidant systems [43]. The free radical scavenging activity of protein-derived molecules is influenced by the amino acid composition because hydrophobic amino acids have superior radical scavenging activity in comparison to hydrophilic amino acids [19]. Indeed, hydrophobic amino acid residues can increase the hydrophobic interactions with membrane lipid bilayers where they are able to exert a significant capacity of scavenging radicals [43,44]. Our results on the reduction in intracellular ROS in cells treated with BPHs can be due to the high concentration of hydrophobic amino acids, such as alanine, proline, leucine, tyrosine, and phenylalanine, found in BPHs. In addition, the presence of other amino acids, i.e., tyrosine, methionine, and tryptophan, known for their antioxidative bioactivity could contribute to the cytoprotective role of BPHs. Moreover, the free radical scavenger capacity also depends on the protein molecular weight distribution, and low-molecular-weight peptides could scavenge free radicals more efficiently than their higher-molecular-weight counterparts [44]. The reduced intracellular ROS production found in BPH-treated cells, could be attributed to peptide patterns less than 25 kDa generated by a hydrolysis reaction and visualized in the SDS-PAGE analysis. However, it cannot be excluded that the observed antioxidant effects in challenged cells are due to the cooperation of other unknown or unidentified antioxidants in our BPHs participating in the protective action against oxidative stress. 

The molecular mechanism underlying the cytoprotective effects of BPHs may involve Nrf2, a transcription activator of antioxidant genes that has been shown to control the expression of key components of the cellular antioxidant system, as well as enzymes involved in ROS and xenobiotic detoxification, and heme metabolism, thus playing a fundamental role in maintaining the redox homeostasis of the cell [45]. In this study, a significant increase in Nrf2 expression after the treatment with BPHs evaluated at both the gene expression and protein levels was observed. Cells treated with BPHs are able to express the *nrf2* gene and to synthesize its corresponding protein to a greater extent than the control. Moreover, the nuclear translocation of the Nrf2 protein evaluated by subcellular localization suggests that the BPH treatment enhances the antioxidant response of cells against stress induced by hydrogen peroxide resulting in a cytoprotective action. Further studies already underway are needed to evaluate the expression of genes involved in the antioxidant response in order to confirm the role of hydrolysates in this cellular defense mechanism and the role of Nrf2 in activating the antioxidant response in cells. 

Over the last decade, the literature has demonstrated that BSF larvae represent a sustainable and high-value source of proteins for animal and aquafeed; given that the 2017 EU regulations allow for BSF protein meal to be used in aquaculture as fish meal substitution and research has proven the nutritional potential, BSF larvae-based derivatives possess peculiar properties, also in promoting animal health, making them precious ingredients in feed formulation. A recent study demonstrated that fish meal exhibits a mild antioxidant behavior compared to BPH in neutrophil cells [19]; moreover, a different modulation of immune response in a fish model organism was also showed in our previous study [3]. This aspect represents a relevant chance for the aquaculture sector that is often subjected to incremental costs as a result of drugs and nutritional supplements usage for fish to cope with common pathogen infections. 

Even though insects have been recognized as a sustainable nutritional source, safety concerns are currently limiting the use of whole insects or their derivatives in many Western countries. Further studies are needed to increase the consumer acceptability of insect-based food. 

Taken together, the results from this study shed light into the biological activity of the BSF larvae protein hydrolysates, herein produced and characterized, and their beneficial effects in preventing oxidative stress in cells. The unique and valuable bioactivities together with data on the safety aspects of using insect-based ingredients as functional and natural additives in diverse industrial fields, ranging from feed to pharmaceutics, could increase the consumer acceptance of insects to feed the world’s growing population. This perspective is extremely fascinating for the great BSF larvae potential in a sustainable waste valorization and management approach within a circular economy.

## 5. Conclusions

Among insect species, the BSF *H. illucens* has attracted particular attention for its great potential in waste valorization while producing a valuable larval biomass with a great potential as a sustainable source of nutrients, including high-value proteins that can be used in pet food and aquaculture feed formulations. In this study, the protein hydrolysates obtained from BSF larvae proteins by enzymatic digestion in a large-scale process were characterized and assessed for their possible biological activity in L-929 cells. Our findings show that the obtained hydrolysates possess a significant antioxidant activity by reducing intracellular ROS in challenged cells and activating the expression and nuclear translocation of Nrf2, a master regulator of the cellular stress response. Overall, the remarkable antioxidant properties strongly encourage the use of these BSF protein hydrolysates in feed formulation as potential health-promoting ingredients. 

## Figures and Tables

**Table 1:** Measured yields with respect to the total dry matter of the main fractions extracted with the procedure.

	Measured Weight (g)	Yield Dry Matter (%)	Yield Total Matter (%)
Water-soluble species	126.40	30.83	12.99
Extracted water-soluble amino acids	85.89	20.95	8.83
*n*-Hexane-soluble species (fat fraction)	83.40	20.34	8.57
Insoluble solid (polymers–chitins)	150.30	36.66	15.45

**Table 2:** Quantification of metabolites obtained with the large-scale production, with specific regard to the free aminoacidic composition. Different quantification units are displayed for an easy comparison with other similar reports.

Compound/Specie	Code Name	Concentration in 1.6 L Solution (mM)	Relative % in Weight for FAAs	Relative % of FAA Particles	W/W of Raw Larvae (mg/g)	W/W of Dry Matter (mg/g)	Total Millimoles in the 1.6 L Solution
Leucine	LEU	32.8 ± 0.5	8.0	7.2	7.1	16.8	52.6
Isoleucine	ILE	35.6 ± 0.6	8.7	7.8	7.7	18.2	56.9
Valine	VAL	43.1 ± 0.8	9.4	9.4	8.3	19.7	68.9
Threonine	THR	21.7 ± 0.7	4.8	4.7	4.2	10.1	34.6
Lactate	LAC	23.6 ± 0.5	3.9	5.2	3.5	8.3	37.7
Alanine	ALA	80.7 ± 0.8	13.4	17.7	11.8	28.0	129.2
Proline	PRO	36.4 ± 0.3	7.8	8.0	6.9	16.3	58.2
Glutamate	GLU	47.1 ± 1.0	12.9	10.3	11.4	27.0	75.4
Methionine	MET	12.4 ± 0.3	3.4	2.7	3.0	7.2	19.9
Aspartate	ASP	0.7 ± 0.1	0.2	0.2	0.2	0.4	1.1
Serine	SER	12.0 ± 0.4	2.3	2.6	2.1	4.9	19.1
Phenylalanine	PHE	18.0 ± 0.6	5.5	3.9	4.9	11.6	28.8
Tryptophan	TRP	8.1 ± 0.2	3.1	1.8	2.7	6.5	13.0
Tyrosine	TYR	15.9 ± 0.8	5.4	3.5	4.7	11.2	25.4
Glycine	GLY	40.6 ± 0.5	5.7	8.9	5.0	11.9	65.0
4-aminobutytrate	GABA	28.6 ± 0.7	5.5	6.2	4.8	11.5	45.7
Ethanol	EtOH	28.3 ± 0.5	-	-	2.1	5.1	45.2
Propanediol	PRDO	10.7 ± 0.4	-	-	1.3	3.2	17.1
Acetate	AcO	92.3 ± 1.5	-	-	9.1	21.6	147.7
Succinate	SUC	55.8 ± 1.5	-	-	10.8	25.7	89.3
3-Hydroxypropanoate	3-HPRO	11.8 ± 0.3	-	-	1.8	4.2	18.9
Dimethylamine	DMA	0.5 ± 0.2	-	-	0.0	0.1	0.8
Trimethylamine	TMA	3.9 ± 0.5	-	-	0.4	0.9	6.3
Ethanolamine	ETN	12.1 ± 1.8	-	-	1.2	2.9	19.4
Choline	CHO	5.6 ± 0.6	-	-	1.0	2.3	8.9
Betaine	BETA	24.9 ± 1.0	-	-	4.8	11.4	39.9
Glycerol	GLYOH	28.5 ± 1.4	-	-	4.3	10.2	45.5
Uracil	URA	4.1 ± 0.5	-	-	0.7	1.8	6.5
Tyramine	TYM	14.9 ± 1.0	-	-	3.4	8.0	23.9
Formate	FOR	27.8 ± 1.0	-	-	2.1	4.9	44.5
Total	TOT	778.5 ± 6.2	-	-	131.3	311.6	1245.5
Total aminoacidic composition	TOT FAAs	457.3 ± 1.8	100.0	100.0	88.3	209.5	731.5

## Data Availability

All data obtained from this study are included in the manuscript.

## Supplementary Materials

The following supporting information can be downloaded at: https://www.mdpi.com/article/10.3390/antiox12020519/s1, Figure S1: ^1^H-NMR spectrum run with NOESY presaturation of the water signal (NOESYpresat). Table S1: Specific assignments allowing quantification after MARA-NMR procedure.
