# Supplementary material for: Cytoprotective and Antioxidant Effects of Hydrolysates from Black Soldier Fly (Hermetia illucens)
Source: Antioxidants (Basel). 2023 Feb 18;12(2):519. doi: 10.3390/antiox12020519 (PMC9952651; doi:10.3390/antiox12020519)
Supplement: Supplementary file 1 [file antioxidants-12-00519-s001.zip › antioxidants-2212962-supplementary.pdf]

*antioxidants*

**Figure S1**. $^{1}$H-NMR spectrum run with NOESY presaturation of the water signal (noesypresat).

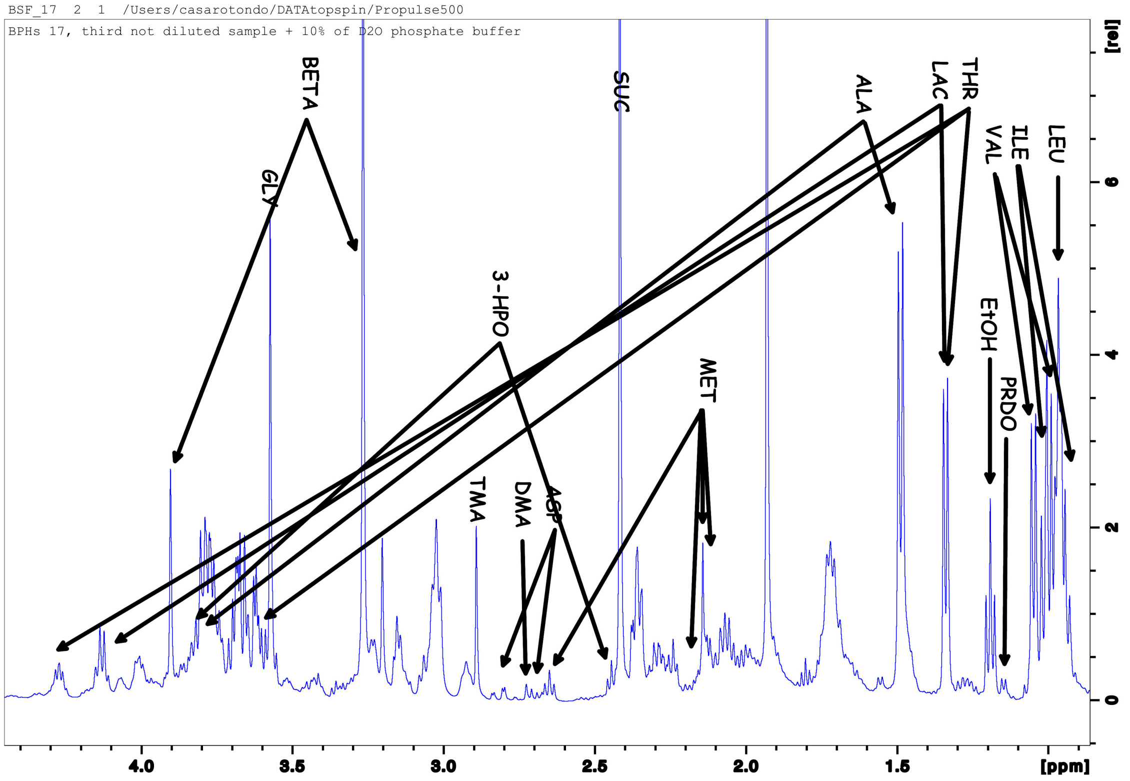


This region (1–4 ppm) shows the main assignment of the crucial peaks. The subsequent integration allowed the quantification of the described 31 metabolites. The $^{1}$H-NMR spectrum for with the assignment pointing to specific metabolites along with other data concerning the assignment and overlapped assignments was later resolved through the MARA-NMR algorithm.

**Table S1**. Specific assignments allowing quantification after MARA-NMR procedure.

| Extended names | Assignment code | Number | Integration limits | | Integration values |
|---|---|---|---|---|---|
| | TSP reference | int_1 | 0.048 | -0.042 | 10.000 |
| leucine | 9/4*LEU | int_2 | 0.949 | 0.902 | 88.571 |
| isoleucine | 3/4*LEU+6*ILE | int_3 | 0.983 | 0.949 | 236.679 |
| valine | 3*VAL+3/2*LEU | int_4 | 1.015 | 0.983 | 170.178 |
| | 3/2*LEU | int_5 | 1.031 | 1.015 | 45.817 |

| | | | | | |
|---|---|---|---|---|---|
| valine | 3*VAL | int_6 | 1.072 | 1.031 | 131.241 |
| | propylenglycol | int_7 | 1.162 | 1.132 | 10.499 |
| ethanol | 3*EtOH | int_8 | 1.218 | 1.162 | 84.766 |
| | 1*ILE | int_9 | 1.317 | 1.218 | 35.898 |
| lactate + threonine | 3*LAC+3*THR | int_10 | 1.362 | 1.317 | 135.917 |
| alanine | 3*ALA+1*ILE | int_11 | 1.522 | 1.422 | 277.710 |
| | butyrrate | int_12 | 1.580 | 1.523 | 22.841 |
| | 1*LEU | int_13 | 1.676 | 1.588 | 60.305 |
| | 2*LEU+ macrostructures | int_14 | 1.772 | 1.676 | 234.713 |
| propandiol | PRDO | int_15 | 1.838 | 1.772 | 35.573 |
| 4-aminobutyrrate | 2*GABA | int_16 | 1.913 | 1.858 | 57.901 |
| acetate | 3*AcO | int_17 | 1.954 | 1.913 | 277.032 |
| proline + glutamate | 3*PRO+1*GLU+1*ILE | int_18 | 2.109 | 1.954 | 203.081 |
| methionine | 1*GLU+5*MET | int_19 | 2.210 | 2.109 | 98.692 |
| | 1*VAL+2*4-HBU+3*ACO+2*GABA | int_20 | 2.330 | 2.210 | 109.181 |
| | 2*GLU+1*PRO | int_21 | 2.388 | 2.330 | 123.570 |
| succinate | 4*SUC | int_22 | 2.430 | 2.388 | 223.131 |
| 3-hydroxypropionate | 3-HPRO | int_23 | 2.470 | 2.430 | 23.670 |
| | 2*MET | int_24 | 2.672 | 2.617 | 9.437 |
| aspartate | 2*ASP+6*DMA | int_25 | 2.741 | 2.672 | 3.911 |
| dimethyl-amine | 6*DMA | int_26 | 2.777 | 2.750 | -1.366 |
| | 2*ASP | int_27 | 2.849 | 2.778 | 1.509 |
| trimethyl amine | 9*TMA | int_28 | 2.901 | 2.863 | 35.449 |
| macromolecular tyramine | | int_29 | 2.963 | 2.901 | 30.591 |
| macromolecular system | | int_30 | 3.058 | 2.963 | 155.646 |
| | unknown | int_31 | 3.095 | 3.058 | 21.668 |

| | | | | | |
|---|---|---|---|---|---|
| | 2*ETN+1*PHE+2gaba? | int_32 | 3.175 | 3.095 | 67.802 |
| | 9*CHO | int_33 | 3.215 | 3.175 | 50.039 |
| | 2*TYM macro | int_34 | 3.248 | 3.215 | 45.902 |
| betaine | 9*BETA | int_35 | 3.288 | 3.248 | 223.646 |
| | 1*PHE+1*PRO+1*TRP | int_36 | 3.379 | 3.288 | 35.039 |
| | 1*PRO | int_37 | 3.446 | 3.379 | 21.689 |
| | unknown | int_38 | 3.492 | 3.446 | 11.625 |
| | 2*TRP | int_39 | 3.536 | 3.492 | 17.329 |
| | 2*GLY+2*0.71*GLYOH | int_40 | 3.584 | 3.543 | 121.686 |
| | 2*0.29*GLYOH+2*4-HBU+1*THR | int_41 | 3.615 | 3.584 | 48.560 |
| | 1*VAL | int_42 | 3.637 | 3.615 | 53.728 |
| | 2*GLYOH+2*EtOH+1*ILE | int_43 | 3.693 | 3.637 | 149.765 |
| | 2*3-HPRO | int_44 | 3.721 | 3.693 | 40.847 |
| | 1*ALA+1*GLU+1*GLYOH+LEU+2*PRDO | int_45 | 3.813 | 3.721 | 264.094 |
| | 2*ETN+1*MET+1*SER | int_46 | 3.879 | 3.813 | 78.949 |
| | 2*BETA+1*ASP | int_47 | 3.933 | 3.879 | 75.667 |
| | unknown | int_48 | 3.983 | 3.933 | 23.577 |
| | 1*PHE+unknown | int_49 | 4.042 | 3.983 | 44.252 |
| | 2*CHO+1*TRP | int_50 | 4.092 | 4.042 | 19.854 |
| | 1*LAC+1*PRO | int_51 | 4.162 | 4.092 | 53.595 |
| | 1*THR | int_52 | 4.216 | 4.162 | 8.731 |
| | | int_53 | 4.324 | 4.224 | 33.728 |
| | | int_54 | 5.839 | 5.753 | -3.059 |
| uracyl | 1*URA | int_55 | 6.456 | 6.355 | 4.244 |
| | 2*TYM+2*TYR | int_56 | 6.965 | 6.815 | 56.014 |
| | | int_57 | 7.130 | 7.083 | 1.877 |
| | 2*TYM+2*TYR+1*TRP | int_58 | 7.250 | 7.130 | 71.203 |
| | 1*TRP | int_59 | 7.299 | 7.251 | 11.665 |
| | 1*TRP+1*PHE | int_60 | 7.354 | 7.300 | 40.250 |
| | 1*PHE | int_61 | 7.403 | 7.354 | 21.238 |
| | 2*PHE | int_62 | 7.467 | 7.403 | 34.346 |

| | | | | | |
|---|---|---|---|---|---|
| | 1*TRP+1*URA | int_63 | 7.577 | 7.489 | 8.069 |
| | 1*TRP | int_64 | 7.762 | 7.684 | 5.313 |
| | | int_65 | 7.877 | 7.843 | 0.649 |
| | | int_66 | 7.949 | 7.877 | 2.607 |
| | | int_67 | 8.244 | 8.172 | 3.722 |
| | 1*FOR | int_68 | 8.507 | 8.428 | 27.793 |

Assignment inferred by a) comparison of the spectral profile with databases fitting both chemical shifts and fine structures and b) 1D TOCSY confirming the specific spin system.
